# The resistance of the yeast *Saccharomyces cerevisiae *to the biocide polyhexamethylene biguanide: involvement of cell wall integrity pathway and emerging role for YAP1

**DOI:** 10.1186/1471-2199-12-38

**Published:** 2011-08-19

**Authors:** Carolina Elsztein, Rodrigo M de Lucena, Marcos A de Morais

**Affiliations:** 1 Interdepartmental Research Group in Metabolic Engineering. Av. Moraes Rego, 1235, Cidade Universitária, 50670-901, Recife, PE, Brazil; 2 Department of Genetics, Federal University of Pernambuco. Av. Moraes Rego, 1235, Cidade Universitária, 50670-901, Recife, PE, Brazil

## Abstract

**Background:**

Polyhexamethylene biguanide (PHMB) is an antiseptic polymer that is mainly used for cleaning hospitals and pools and combating *Acantamoeba *infection. Its fungicide activity was recently shown by its lethal effect on yeasts that contaminate the industrial ethanol process, and on the PE-2 strain of *Saccharomyces cerevisiae*, one of the main fermenting yeasts in Brazil. This pointed to the need to know the molecular mechanism that lay behind the cell resistance to this compound. In this study, we examined the factors involved in PHMB-cell interaction and the mechanisms that respond to the damage caused by this interaction. To achieve this, two research strategies were employed: the expression of some genes by RT-qPCR and the analysis of mutant strains.

**Results:**

Cell Wall integrity (CWI) genes were induced in the PHMB-resistant *Saccharomyces cerevisiae *strain JP-1, although they are poorly expressed in the PHMB-sensitive *Saccharomyces cerevisiae *PE2 strain. This suggested that PHMB damages the glucan structure on the yeast cell wall. It was also confirmed by the observed sensitivity of the yeast deletion strains, *Δslg1, Δrom2, Δmkk2*, *Δslt2*, *Δknr4*, *Δswi4 *and *Δswi4*, which showed that the protein kinase C (PKC) regulatory mechanism is involved in the response and resistance to PHMB. The sensitivity of the *Δhog1 *mutant was also observed. Furthermore, the cytotoxicity assay and gene expression analysis showed that the part played by *YAP1 *and *CTT1 *genes in cell resistance to PHMB is unrelated to oxidative stress response. Thus, we suggested that Yap1p can play a role in cell wall maintenance by controlling the expression of the CWI genes.

**Conclusion:**

The PHMB treatment of the yeast cells activates the PKC1/Slt2 (CWI) pathway. In addition, it is suggested that HOG1 and YAP1 can play a role in the regulation of CWI genes.

## Background

Previous work has shown that polyhexamethylene biguanide (PHMB) was effective in selectively killing the yeasts that are regarded as contaminants of the ethanol fermentation process, with special attention being paid to *Dekkera bruxellensis *[1]. PHMB has been used as an antiseptic and disinfectant in medicine and industry, and its current applications include the following: impregnation of fabrics to inhibit microbial growth [2,3], water treatment [4], disinfection of a wide range of solid surfaces such as contact lenses [5], mouthwashing [6,7], the treatment of hatching eggs to prevent contamination by *Salmonella *[8,9], treatment against fungi [10] and *Acanthamoeba *[11-13]. Its commercial preparations consist of mixtures of polymeric biguanides of blocks with molecular structure [-CH2)_6_.NH.C(= NH).NH.C(= NH).NH-]_n_, where n may vary from 2 to 40 (average 11) and molecular mass ranging from 400 to 8000 [14].

The heterogeneity of the molecule is increased further by the presence of either amine, or cyanoguanidine or guanidine end-groups in any combination at the terminal positions of each chain. Nonetheless, the cationic nature of the guanidine group makes the molecule positively charged at physiological to low pH [15]. As a result, PHMB is highly adsorptive to anionic surfaces such as bacterial cell walls and its biocidal mechanism may include damage to the cell membrane [16]. At lower concentrations, it has been suggested that the bacteriostatic effects of PHMB may be partly due to a powerful cooperative interaction with (polyanionic) nucleic acids [17]. It was suggested that PHMB should interact with phospholipids of the *Acanthamoeba *cell membrane and thus cause changes in cell permeability [18,19], and this was further supported by work on *E. coli *[14]. The external layer of *Saccharomyces cerevisiae *and other yeast species plasma membrane is enhanced by phosphatidylcholine, ergosterol and sphingolipids [20]. Owing to the cationic nature of PHMB, its toxic effect may be mediated by its links to the negative phospholipids on the yeast cell surface. In the presence of PHMB, the homogeneous distribution of phospholipids that are usually linked to biological membranes, is transformed into a mosaic of individual phospholipid domains that produce fluid and liquid crystalline regions in the cell membrane [15]. By means of the whole-genome transcriptional profile, Allen et al [14] show that *E. coli *responds to bacteriostatic levels of PHMB by altering the expression of many of the genes involved at all levels of the cell ultra structure, i.e. the outer membrane, periplasm, inner membrane and cytoplasmic domains, but not the lipolysaccharide layer. There was also an alteration in the expression of genes associated with stresses such as acid resistance, alkali resistance, osmotic shock and cell-envelope perturbation.

As well as the fungicide activity in *D. bruxellensis*, *Pichia galeiformes *and *Candida tropicalis*, the yeast species isolated PHMB as a contaminant through ethanol fermentation, and this also differentially affected *Saccharomyces cerevisiae *industrial strains, for example the PE-2 strain was more affected than JP1 [1]. This result might impose constraints on the use of PHMB in controlling incidents of yeast contamination in industrial fermentations. Thus, the further steps towards developing industrial formulations of PHMB, must rely on determining the identification of biological activity and targets of PHMB on the yeast cells, and identifying the yeast mechanisms that respond to the damage caused by this biocide.

In this study, we have sought to understand the aspects of PHMB-cell interaction discussed above, by adopting two strategies: 1) testing the biocide effects of PHMB on the expression of yeast genes involved in the cell wall integrity mechanism (CWI) and 2) by testing yeast strains with mutations in genes involved in CWI and their oxidative stress response. The results strongly indicated that PHMB may act by destabilizing the yeast cell wall, thus inducing the cell wall integrity pathway response. Moreover, an account is given of the potential regulatory role of Yap1p on the expression of CWI genes.

## Results and Discussion

### PHMB treatment induces genes of the yeast Cell Wall Integrity (CWI) sensing mechanism

**Figure 1:**
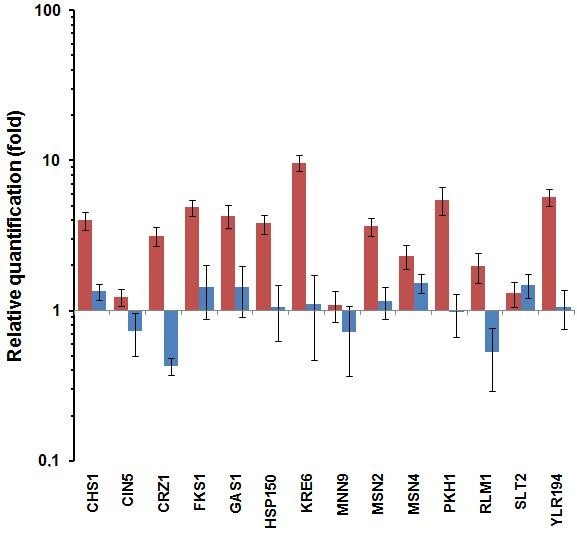
**Relative expressions of the yeast genes involved in the cell wall integrity mechanism and in the general stress response**. Relative Quantity represents the expression of the studied genes in the PHMB-treated cells (0.01%) over non-treated cells of the two industrial strains JP1 (red column) and PE-2 (blue column).

Our previous results showed that the lethal effect of PHMB on yeast cells was partly mitigated by trehalose [1], which is known to be a protective agent of the cell envelope [20]. Moreover, previous global gene expression analysis revealed that exposure of *E. coli *cells to PHMB induced the expression of genes involved in cell wall maintenance [14]. This led us to search for CWI genes in the yeast genome (Additional file 1). Moreover, recent studies have shown that some genes involved in trehalose metabolism are regulated by stress response elements (STRE) in their promoter region; [21], in view of this we have included some yeast genes containing STRE in their promoter in our analysis. From a list of selected genes (Additional file 1), we conducted a quantitative PCR analysis after cell exposure to PHMB and the results revealed that the *CHS1*, *FKS1, GAS1*, *HSP150*, *KRE6*, *MSN2, MSN4, PKH1 *and *YLR194c *genes were up-regulated in the PHMB-resistant JPI strain, while remaining unchanged in the PHMB-sensitive PE-2 strain (Figure 1). Moreover, *CRZ1 *and *RLM1 *genes were induced in JP1 and repressed in PE-2, while *CIN5 *and *MNN9 *did not change in JP1, but were repressed in PE-2. The expression of the *SLT2 *gene remained unchanged in both the industrial strains (Figure 1).

The products of the *CHS1, GAS1, FKS1*, *HSP150*, *KRE6, PKH1 *and *YLR194c *genes can be located either in the cell membrane or in the cell wall, and are mostly involved in the maintenance and remodeling of the cell envelope in response to environmental changes. The membrane-located chitin synthase 1, encoded by *CHS1*, acts on the turn-over of the chitin in the septum during the cell division [22]. The *GAS1 *gene product is a GPI-anchored protein located in the plasma membrane that acts by stretching the cover of β-1,3-glucan during the remodeling of the cell wall. Its expression is dependent on the SBF transcription factor, which comprises the Swi4p and Swi6p proteins [23]. The *FKS1 *gene encodes the catalytic sub-unit of the 1,3-β-D-glucan synthase complex. This complex is controlled by the regulatory subunit Rho1p, which in turn is activated by the nutrient sensing TOR pathway that regulates the activity of protein kinase C - Pkc1p, a key component of the Cell Wall Integrity (CWI) mechanism [21]. The Hsp150p protein is located in the cell wall and is secreted into the medium in response to heat or osmotic shock. Its gene is up-regulated under Calcofluor white and Zymolyase [24], heat shock [25] nitrogen limitation [26] treatments and by decreasing the β-1,3-glucan level in the cell wall [27]. The *PKH1 *gene encodes a phosphoinositol-dependent protein kinase, whose function overlaps Pkh2p, and is involved in maintaining the cell wall. This protein phosphorylates and activates Pkc1p [28]. The ORF *YLR194c *encodes for a putative GPI-attached protein and its expression is induced by cell wall stress or by mutation in the *FKS1 *gene [29]. The *MNN9 *gene encodes a β-1,6-mannosyltransferase involved in the transfer of mannose from UDP-mannose to oligosaccharides during the synthesis of the β-glucan bridge of the cell wall [30]. In addition, the *KRE6 *gene encodes a putative synthetase that acts on the synthesis of the β-1,6-glucan cover and mutation in this gene causes a 50% reduction in the synthesis of β-1,6-glucan in the cell wall [29]. The Kre6p protein was shown to be phosphorylated and genetically interact with components of the Pkc1p-mediated MAP kinase pathway [31] in order to regulate the β-1,6-glucan synthesis [32]. It was found that over-expression of the *KRE6 *gene offset the defect in the cell wall organization of the *pkc1*-mutant [28]. Thus, this gene could be a key to explain the difference in resistance of the two industrial strains to PHMB, since it was highly expressed in the PHMB-resistant JP1 strain (Figure 1).

Crz1p is a calcineurin-dependent zinc finger- type transcription factor that binds to the promoters of the *FKS1 *and *CHS1 *genes in response to calcofluor and calcium, respectively [23]. Both genes were found to be co-expressed, with a peak in G1, when the isotropic cell wall synthesis allowed daughter-cell expansion [23] and our results (Figure 1) corroborate that of the co-expression pattern. The lower expression level of the *CRZ1 *gene in PE-2 could explain the lack of *CHS1 *and *FKS1 *induction in this strain, and together with the low expression of *KRE6*, could produce the previously mentioned sensitive phenotype of the PE-2 strain to PHMB [1]. Moreover, these results suggest that the tolerance of the JP1 strain might be due to a more effective restoration of the cell envelope after it has been damaged by PHMB. This might be evidence that PHMB damages the glucan structure on the yeast cell wall.

### Resistance to PHMB depends on the functionality of the PKC and HOG mechanisms

**Figure 2:**
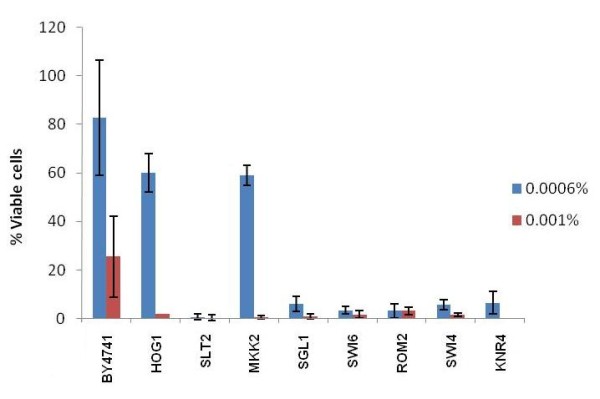
**Cytotoxicity assay of yeast CWI mutants to different doses of PHMB**. The percentage of viable cells after treatments refers to the number of CFU in treated samples over compared with non-treated samples of a given strain.

To test the hypothesis of cell wall damage, resistance to PHMB was evaluated in yeast strains with deletion in a series of CWI genes, including the genes involved in the HOG and PKC pathways (Additional file 2). The results of the cytotoxicity assay showed that deletion of the *SLG1/WSC1, SLT2, ROM2, SWI4*, *SWI6 *and *KNR4 *genes impaired the chances of cell survival to exposure to PHMB, while deletion of *HOG1 *and *MKK2 *genes only affected cell survival at higher PHMB concentration (Figure 2). The remaining mutants tested did not show altered sensitivity to this compound (data not shown). *SLG1/WSC1 *gene encodes one of the sensor-transducer proteins located in the cell membrane that activates the GDP/GTP exchange protein Rom2p in response to cell wall damage [28]. It was observed that the absence of this protein impairs the activation of Slt2p/Mpk1p kinase by heat shock [33]. The hypersensitivity of the *Δslg1/wsc1 *mutant to PHMB (Figure 2), together with the parental phenotype of yeast mutants for other sensors (Additional file 2), showed that Slg1p/Wsc1p is the main sensor for the damage caused by PHMB.

Following the PKC cascade, it was observed that the *Δrom2 *mutant was very sensitive to PHMB (Figure 2). The GTP/GDP-exchange factor Rom2p activates the Ras-like protein kinase Rho1p that triggers the PKC cascade by activating Pkc1p, which in turn activates Bck1p [23]. Bck1p, known as MAP kinase kinase kinase (MAPKKK), phosphorylates Mkk2p (MAPKK) in response to cell wall damage, which further phosphorylates Mpk1p/Slt2p (MAPK). The parental phenotype of the *Δbck1 *mutant and the intermediary sensitivity of the *Δmkk2 *mutant to PHMB (Figure 2) could be explained by the complementation by their paralogous Bck2p and Mkk1p, respectively.

Furthermore, the hyper-sensitivity of the *Δmpk1/slt2 *mutant (Figure 2) showed the importance of the *SLT2 *gene in PHMB resistance. Slt2p resides predominantly in the nucleus under non-stress conditions, but rapidly relocates to the cytoplasm in response to cell wall stress [34]. Once activated, Slt2p phosphorylates and activates the Rlm1p [23] transcription factor, as well as Swi4p [28]. As stated above, Swi4p and Swi6p form the SBF transcriptional factor that regulates the CWI genes, such as *GAS1, FKS1 *and *SMI1/KNR4 *[23]. Mutation of the *SMI1/KNR4 *gene increased the sensitivity of the yeast cells to PHMB (Figure 2). It was recommended that Smi1p/Knr4p should coordinate the cell cycle progression in connection with cell wall synthesis and this protein was shown to be essential for viability of the cell in the absence of a functional PKC1/Slt2 pathway [35]. A physical interaction between Knr4p and Slt2p was also described and it was proposed that such interaction modulate the Slt2p-dependent activation of Rlm1 and SBF transcriptional factors [36]. Activation of Rlm1p induces the expression of *CHS1, FKS1, KRE6 *and *HSP150 *[23], the same genes up-regulated in JP1 strain upon the PHMB treatment (Figure 1).

Finally, the *Δhog1 *mutation showed intermediate sensitivity to PHMB (Figure 2). It was suggested that at hyperosmotic shock, Hog1p activates Rlm1p that in turn regulates the subsequent expression of the *SLT2 *gene [37]. Thus, a cross-talk between HOG and PKC MAPK pathways should be established following the perturbation of the cell wall. The results of this research indicate that Hog1p may act as an amplifier of the sensing mechanism of the cell wall damage caused by PHMB, as has been recently described in the case of other cell wall damage [38]. Furthermore, double mutant analysis showed that there was a synergistic interplay between Hog1p and Yap1p in a resistance to arsenite stress, which suggests the existence of a genetic inter-dependence of both genes on the stress response [39]. Recent studies have shown that Hog1p is involved in cell wall remodeling by regulating the expression of *EXG1 *gene, which encodes the glucanase that affects the level of β-glucan [38]. This hypothesis supports the idea that PKC and HOG pathways converge to regulate the maintenance of the cell wall [38,40,41]. On the basis of this evidence, we suggest that PHMB may affect the β-glucan structure of the cell wall that is sensed by the PKC pathway, together with Hog1p.

### *The YAP1 *gene is also involved in the cellular resistance to PHMB, but not through oxidative stress response

**Figure 3:**
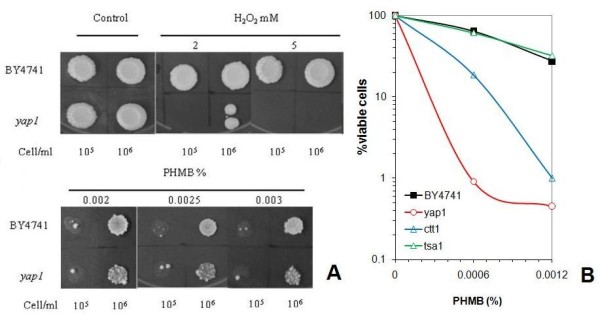
**Cytotoxicity assay to PHMB of yeast strains with deletion in oxidative stress response genes**. **(A) **Spot test assay of parental BY4741 and *Δyap1 *mutants to oxidizing compound H_2_O_2 _and to PHMB. **(B) **Dose-response cell survival in a PHMB assay of *ctt1*, *tsa1 *and *yap1 *mutant strains.

**Figure 4:**
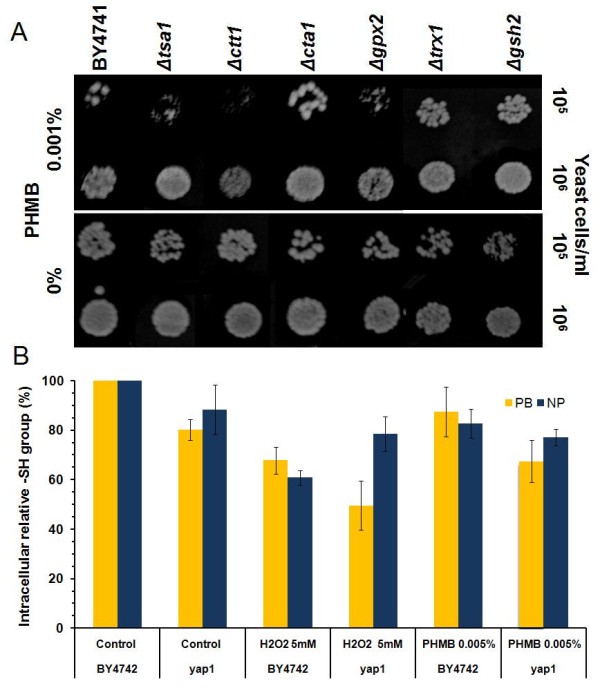
**Cytotoxicity assay to PHMB of yeast strains with deletion in oxidative stress response genes**. **(A) **Spot test assay in YPD plates in the presence and absence of PHMB. **(B) **Determination of relative sulfhydryl groups in soluble proteins (PB) and non-proteins (NP) of molecules in the cell extract of BY4741 parental yeast cells and *Δyap1 *mutant strains in the presence of 0.005% PHMB and 5 mM H_2_O_2_.

Following the induction of the *MSN2 *and *MSN4 *genes in the JP1 strain upon exposure to PHMB (Figure 1), we further screened the collection of deletion strains whose genes encode proteins involved in the complex general stress response mechanism (Additional file 2). Among these, the *Δyap1 *mutant showed a high degree of sensitivity to PHMB (Figure 3A, B). It was suggestive of the induction of oxidative damage to the cell membrane by PHMB. The Yap1p-mediated regulatory pathway that controls the oxidative stress adaptive response is activated by redox sensory mechanisms that detect changes in the intracellular redox balance caused by reactive oxygen species (ROS) and by oxidized thiols such as glutathione, thiorredoxin and SH-containing proteins [42]. Sulfhydryl groups, including non-protein (NP-SH), mostly represented by glutathione, and protein bound (PB-SH), are abundant in cells and can be oxidized by ROS [43]. Nevertheless, we did not observe only a small change in the mobilization of intracellular thiol groups when BY4741 or *yap1 *strains are submitted to PHMB for 72 hours, as it was observed after H_2_O_2 _reference treatment (Figure 4B). Moreover, no increase in the peroxidation of the membrane phospholipids was observed (data not shown). In addition, no other strain with deletion in oxidative stress genes was sensitive to PHMB, except the *Δctt1 *mutant (Figure 3B, 4A). The *CTT1 *gene encodes the cytosolic catalase T involved in the breakdown of hydrogen peroxide to oxygen and water, which is regulated by the oxidative stress transcription factors Yap1p and Skn7p and by the general stress response transcriptional complex Msn2/4p [44]. Winderickx et al. [45] found that oxidative stress-induced expression of *CTT1 *requires Yap1p even though this gene does not include a YRE motif in its promoter, although it contains a STRE motif [45]. Yap1p participates in the regulation of both antioxidant genes and several drug resistance genes [46-48], and this transcription factor regulates the genes that do not have YRE in their promoter regions [49,50]. Yap1p plays a role in regulating the expression of components of the STRE activation machinery, and as a result, the STRE-dependent gene expression, even though Yap1 does not bind STREs directly [49]. This suggests that *YAP1 *and *CTT1 *genes might be involved in the resistance to PHMB in a way that is not linked to the oxidative stress response pathway. There are some indications of the participation of the upper elements of the PKC pathway in the survival of cells when subjected to oxidizing agents such as diamide and hydrogen peroxide [51]. It is known that ethanol induces the expression of various stress responsive genes, such as *CTT1*, through the binding of the Msn2/4 transcriptional complex to their promoters. This complex is activated by Hog1p phophorylation that leads to the migration of Msn2p and Msn4p from the cytosol to the nucleus so that they can trigger the induction of the target genes [52]. This indicates that there is a connection between the CWI pathway (PKC-HOG) and the oxidative stress response in *Saccharomyces cerevisiae*, in which Ctt1p can act as a bridge. Very recently, Liu et al [53] found that *SOD1 *gene is involved in tolerance to cell wall damaging agents in yeast, which supports this connection between these pathways.

It was shown that Yap1p appears to be necessary to activate the *SRP1 *gene that encodes a cell wall protein [54]. The *Δyap1 *mutant was reported to be sensitive Congo Red [54], as we also observed (data not shown). Taken together, the evidence points to the involvement of Yap1p in the CWI mechanism.

### Yap1p also regulates the expression of CWI genes

**Figure 5:**
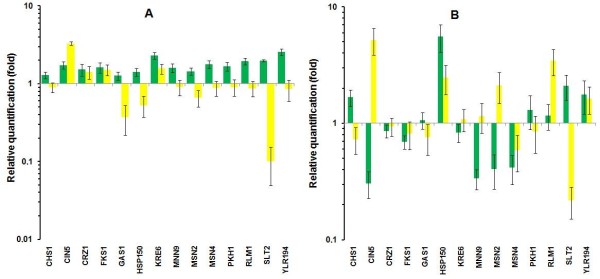
**Relative Quantity of yeast genes involved in the cell wall integrity mechanism and the general stress response of parental BY4741 (green column) and *Δyap1 *(yellow column), strains in response to treatments with 0.005% PHMB (**A**) and heat shock at 42°C (**B**)**.

The possible involvement of Yap1p in the regulation of CWI genes was tested by measuring the expression in parental and *Δyap1 *strains of those genes when they were up-regulated by PHMB in the JP1 strain. The results showed that only *KRE6 *and YLR194c were slightly up-regulated by PHMB treatment in the parental BY4741 cells (Figure 5A), which could explain the high sensitivity of this laboratory strain compared to JP1 (data not shown). Two results were particularly relevant in the *Δyap1 *mutant. First, the expression of the *SLT2 *gene was down-regulated by a factor of ten (Figure 5A), which was not observed even in the PHMB-sensitive industrial PE-2 strain (Figure 1), and the slight expression of YLR194c was removed (Figure 5A). It is also noteworthy that the *CIN5 *gene showed up-regulation by a factor of 3.5 after the PHMB treatment (Figure 5A), while no change was observed for this gene in either of the industrial strains (Figure 1). It can be concluded that Yap1p also works in response to cell wall damage by controlling the expression of the genes at the lower part of the PKC cascade. It is known that Slt2p phosphorylates and activates the Rlm1p transcription factor, which in turn back-regulates the expression of the *SLT2 *gene itself [28]. In view of this, Yap1p could be involved in the regulation of both the *RLM1 *and *SLT2 *gene expression. This idea was strengthened by further observation which showed that the *SLT2 *gene was down-regulated in the *Δyap1 *mutant after heat shock treatment (Figure 5B). The behavior of the *RLM1 *gene was opposite to its expression, in which a significant over-expression was observed in the *Δyap1 *mutant after heat shock treatment (Figure 5B). Thus, we suggest that Yap1p should directly co-regulate the expression of the *SLT2 *gene in response to damage in the cell wall. As a result, this protein should work on the co-regulation of CWI genes. This co-regulation can amplify the signal for CWI gene expression. It was reported that activation of Slt2p by heat shock could occur in a PKC-independent manner [55]. Thus, we suggest that this regulation should occur via Yap1p. The presence of TF binding sites in the promoter region of the CWI genes was identified by an *in silico *analysis (Additional file 1 and Additional file 2). The fact that we did not observe the presence of the conserved Yap1-Responsive Element (YRE) motif at the *SLT2 *gene promoter does not impair its regulation by Yap1p. This suggestion is supported by experimental evidence that shows signs of the regulation of STRE-dependent genes by Yap1p, even though this protein does not recognize the STRE motif [49]. All these possible alternatives must be tested further.

## Conclusion

In this study, the up-regulation of a number of genes required for cell wall polymer synthesis and remodeling has been shown by RT-qPCR upon PHMB-treatment of PHMB-resistant *Saccharomyces cerevisiae *industrial JPI strain, but not in the PHMB-sensitive PE-2 strain. This finding indicates the involvement of the CWI pathway in the tolerance to the cell wall stress caused by this biocide. In addition, the up-regulation of the genes encoding Msn2/4 transcription factors indicates that the general stress response could also be involved in yeast cell response to PHMB. A cytotoxicity assay of the wild-type BY4741 strain and a series of mutant strains (with single deletion in CWI genes, in *HOG1 *and *YAP1 *and a number of other genes related to oxidative stress response) confirmed the involvement of the CWI pathway in the response to PHMB cell wall damage. Hog1p should act as one of the modulators of this response. In addition, the products of *YAP1 *and *CTT1 *genes also work on the resistance to PHMB in a way that does not depend on their participation in the oxidative stress response pathway. The fact that *SLT2 *gene expression was down-regulated after PHMB or heat shock treatments in the *Δyap1 *mutant indicates the potential regulatory role of Yap1p in the expression of CWI genes.

## Methods

### Strains and media

Industrial strains of *Saccharomyces cerevisiae *JP1 and PE-2 were described in advance [56]. The yeast mutant strains were taken from the EUROSCARF collection (Institute of Microbiology of the University of Frankfurt, Germany) or kindly provided by Prof Johan Thevelen (Catholic University of Leuven, Belgium) (Additional file 2). The cells were cultivated and maintained in YPD medium (1% yeast extract, 2% peptone, 2% glucose), with 2% agar for solid medium. The antifungal compound PHMB (polyhexamethyl biguanide) was provided by AEB Bioquímica Latino Americanas S/A (Brazil).

### Citotoxicity tests

Yeast cells were pre-cultivated overnight in YPD at 30°C and re-inoculated in the same medium for the exponential growth phase (5 hours) or stationary growth phase (24 hours). The cells were collected by centrifugation, washed in 0.85% NaCl and re-suspended in 100 mM potassium phosphate buffer pH 7 to concentration of 2 × 10^7 ^cells/ml.The yeast cells were treated for 30 minutes in the presence of different concentrations of PHMB with agitation at 180 rpm, and adequately diluted and plated on YPD. After five days of incubation at 30°C, the number of viable cells after each treatment was calculated from the percentage of colony forming units using water-treated cells as the reference of 100% cell viability. The results represent the average of biological replicates with technical triplicates for each. Semi-quantitative spot-test assays were conducted with exponentially growing cells at 2 × 10^7 ^cells/ml in 100 mM potassium phosphate buffer pH 7. After carrying out appropriate dilutions, aliquots of 5 μl were spotted on YPD plates, supplemented with PHMB in different concentrations. The plates were incubated at 30°C for 72 hours.

### Determination of sulfhydryl groups

Protein- bound sulfhydryl (PB-SH) levels were measured in accordance with the method of Sedlak and Lindsay [57], by subtracting the non- protein sulfhydryl (NP-SH) content from the total sulfhydryl (T-SH) content [43]. Cells of the BY4741 and *Δ yap1 *strains were grown on YPD plates in the presence of 0.5 mM H_2_O_2_, 0.005% PHMB and without treatment for 72 hours at 30°C. Approximately 10^9 ^cells were collected from each culture. Protein extracts were obtained in 0.02 M EDTA pH 4.7 with the addition of glass beads, followed by centrifugation at 17 900 *g *for 15 min. The T-SH concentrations were determined by absorption levels at 412 nm after incubating 200 μl aliquots of protein extracts supernatants with 780 μl of 0.2 M Tris pH 8.2 and 20 μl of 5 mM DTNB for 30 min. The (NP-SH) contents were determined in the supernatant, after protein precipitation with 5% trichloroacetic acid (final concentration) by incubating 450 μl supernatant, 900 μl 0.4 M Tris pH 8.9 and 26 μl 5 mM DTNB for 5 min. Absorbance was measured at 412 nm and the protein bound sulfhydryl (PB-SH) content was calculated by subtracting the NP-SH value from the T-SH content. The results are relative to the concentration of these groups in the control cells (100%) that were not exposed to any agent, and represent the average of three separate experiments.

### Cell treatments for gene expression analysis

Two independent cultures of each strain of *Saccharomyces cerevisiae *were grown in YPD until the early stationary phase. The cultures were re-inoculated in fresh medium to an initial DO at 600 nm of 0.5 and split in two flaks for non-treated and PHMB-treated cells at 0.005% (for haploid laboratory strains) and 0.01% (for industrial strains). After incubation for one hour at 30°C with agitation, the cells were collected by centrifugation and immediately frozen in liquid nitrogen and stored at -80°C. For genes response to thermal shock, cells of BY4741 and *Δyap1 *strains prepared as above and test cell suspensions, were incubated for one hour 42°C, while the reference was incubated at 30°C. Following this, the yeast cells were collected and stored as above.

### RNA extraction and cDNA Synthesis

For total RNA extraction, the frozen cells were suspended in 600 μl of AE buffer (50 mM sodium acetate, 10 mM EDTA, adjusted to pH 5.3 with acetic acid), 40 μl of 10% SDS and 600 μl phenol equilibrated to pH 4 (Invitrogen) and homogenized 15 seconds on vortex. Following incubation at 65°C for 10 minutes, with mixing every two minutes, and incubation for five minutes at 4°C, the lysate was centrifuged at 13,000 g for five min at 4°C. The aqueous phase was recovered to a new tube and extracted once with equal volume of phenol-chloroform (5:1) solution pH 5.3 and once with one volume of chloroform. Finally, lithium chloride was added to 2.5 M final concentration and total RNA was precipitated for 30 minutes at -20°C and recovered by centrifugation for 20 min at 13,000 g. The pellet was washed with 70% cold ethanol, dried at room temperature for 10 minutes, suspended in 25 μl of DEPC-treated de-ionized water and stored at -80°C until use. RNA quantification was performed by absorbance analysis at 260 nm (1 OD_260 _= 40 μg RNA/ml). The purity was analyzed by the absorbance ratio 260 nm/280 nm that ranged from 1.9 to 2.1. The integrity of RNA was verified by agarose gel electrophoresis and used the sharpness and intensity of bands corresponding to 26S and 18S rRNA as parameters. For the cDNA synthesis, one μg of total RNA was used for 40 μl of reverse transcription reaction with the kit ImProm-II ™ Reverse Transcription System Promega II (Promega, USA), following the manufacturer's instructions.

### Quantitative RT-PCR (RT-qPCR)

The coding regions of the target genes were recovered from the Yeast Genome Database - SGD http://www.yeastgenome.org. The primer design was undertaken by the on-line Genscript Primer Design in advanced mode http://www.genscript.com/cgi-bin/tools/primer_genscript.cgi using the following parameters: the sizes of the primers between 17 and 25 bases, Tm value of 59°C and size of amplicons between 70 and 110 bp. The primer pairs were analyzed with a Netprimer tool to determine the formation of self-hybrids, duplex, hairpins and loops http://www.premierbiosoft.com/netprimer/netprlaunch/netprlaunch.html and select those with a ranking greater than 90%. The primer pairs were subjected to a matching analysis with coding regions of target genes http://www.blast.org and to PCR in sand http://genome.ucsc.edu/cgi-bin/hgPcr using the genome of *S. cerevisiae *as a template. The primers were synthesized by IDT Technologies (USA). Tests on Real-Time PCR were conducted in the ABI Prism 7300 (Applied Biosystems, Foster City, CA, USA) detection system, using the SYBR Green PCR Master Mix (Applied Biosystems) kit. The amplification conditions adopted were: initial step at 50°C for 2 min, 95°C for 10 minutes, and 40 cycles of 95°C for 15 sec, and 60 min for 1 min. To determine the degree of contamination by genomic DNA, the PCR reactions were carried out with RNA samples for each condition. The results produced no detectable amplification in any condition. The values of the Cq threshold cycle were given automatically for the independent amplification. Raw Cq values for all the samples were then plotted in Microsoft Excel 2007 worksheets to create a suitable input file for geNorm applet that complied with the User's Guide [58] The first step was to determine the expression stability for the candidate reference genes - *PDC1, LEU4, ADK1, ADH3 *and *EFB1 *(Additional file 3). *ADK1 *and *EFB1 *have been described as reference genes [59,60] and the others form a part of the laboratory reference genes collection. According to geNorm, at least two of them should be used for data normalization (pairwise variation equal to 0.11) (Additional file 4) and *ADK1 *and *EFB1 *were chosen on the basis of the M value (Additional file 4 and Additional file 5).

### *In silico *gene promoter analysis

The sequences corresponding to -1000 to -1 nucleotide position of the target genes, were recovered in FAST format from the SGD database and used to search for TF binding sites and DNA motifs at the YEASTRACT (**Y**east **S**earch for **T**ranscriptional **R**egulators **A**nd **C**onsensus **T**racking) database at http://www.yeastract.com/index.php[61] following the instructions in the tutorial, and at the *Saccharomyces cerevisiae *Promoter Database http://rulai.cshl.edu/SCPD.

## Authors' contributions

CE carried out the cytotoxicity and molecular genetic studies and drafted the manuscript. RML conducted all the normalization analysis with the aid of geNorm applet for RT-qPCR. MAMJ conceived the study, and participated in its design and coordination as well as giving assistance in drafting the manuscript. All the authors read and approved the final manuscript.

## Supplementary Material

Additional file 1 ***Saccharomyces cerevisiae *genes tested by RT-qPCR**. List of yeast genes selected from the *Saccharomyces *Genome Database that were tested by RT-qPCR upon cell treatment with PHMB and heat shock. The presence of general stress DNA binding motifs (STRE) and the recognition sequence for the transcription factor Yap1p (YRE) in the promoter region of those genes are shown. Click here for file

Additional file 2 **Mutant **strains **with deletion in genes involved in the Cell Wall Integrity (CWI) mechanism**. List of BY4741-derivative mutant strains with deletion in genes involved in the Cell Wall Integrity (CWI) mechanism, including genes belonging to PKC1 and HOG pathways. The presence of general stress DNA binding motifs (STRE) and the recognition sequence for the transcription factor Yap1p (YRE) in the promoter region of those genes are shown. Click here for file

Additional file 3 **Relative quantity of reference genes**. Relative quantity of reference genes in the presence (test sample) or absence (reference sample) of PHMB and heat shock (HS) used as input data for geNorm analysis. Click here for file

Additional file 4 **Selection of reference genes for RT-qPCR**. Graphics of Average expression stability values of remaining control genes and Determination of the optimal number of control genes for normalization obtained from geNorm analysis of the reference genes tested. Click here for file

Additional file 5 **Normalization factors calculation for RT-qPCR analysis**. Summary of the normalization factors calculation of the reference genes *ADK1 *and *EFB1 *for the biological replicates (experiments 1 and 2) in the presence (test sample) or absence (reference sample) of PHMB and heat shock (HS) used as input data for geNorm analysis. Click here for file
