# Supplementary material for: The resistance of the yeast Saccharomyces cerevisiae to the biocide polyhexamethylene biguanide: involvement of cell wall integrity pathway and emerging role for YAP1
Source: BMC Mol Biol. 2011 Aug 19;12:38. doi: 10.1186/1471-2199-12-38 (PMC3175164; doi:10.1186/1471-2199-12-38)
Supplement: Additional file 1 — Saccharomyces cerevisiae genes tested by RT-qPCR. List of yeast genes selected from the Saccharomyces Genome Database that were tested by RT-qPCR upon cell treatment with PHMB and heat shock. The presence of general stress DNA binding motifs (STRE) and the recognition sequence for the transcription factor Yap1p (YRE) in the promoter region of those genes are shown. [file 1471-2199-12-38-S1.DOC]

## Additional file 1

| **Gene** | **ORF** | **Biological function of the gene^a^** | **STRE^b^** | **YRE^c^** |
| --- | --- | --- | --- | --- |
| *SLT2* | YHR030c | Serine/threonine MAP kinase involved in regulating the maintenance of cell wall integrity, progression through the cell cycle, and nuclear mRNA retention in heat shock; regulated by the PKC1-mediated signaling pathway |  |  |
| *MSN2* | YMR037c | Transcriptional activator related to Msn4p; activated in stress conditions, which results in translocation from the cytoplasm to the nucleus; binds DNA at stress response elements of responsive genes, inducing gene expression | **Yes** |  |
| *MSN4* | YKL062w | Transcriptional activator related to Msn2p; activated in stress conditions, which results in translocation from the cytoplasm to the nucleus; binds DNA at stress response elements of responsive genes, inducing gene expression | **Yes** |  |
| *CRZ1* | YNL027w | Transcription factor that activates transcription of genes involved in stress response; nuclear localization is positively regulated by calcineurin-mediated dephosphorylation | **Yes** |  |
| *RML1* | YPL089c | MADS-box transcription factor, component of the protein kinase C-mediated MAP kinase pathway involved in the maintenance of cell integrity; phosphorylated and activated by the MAP-kinase Slt2p |  |  |
| *FKS1* | YLR342w | Catalytic subunit of 1,3-beta-D-glucan synthase, functionally redundant with alternate catalytic subunit Gsc2p; binds to regulatory subunit Rho1p; involved in cell wall synthesis and maintenance; localizes to sites of cell wall remodeling |  |  |
| *CIN5* | YOR028c | Basic leucine zipper (bZIP) transcription factor of the yAP-1 family, mediates pleiotropic drug resistance and salt tolerance; nuclearly localized under oxidative stress and sequestered in the cytoplasm by Lot6p under reducing conditions |  |  |
| *CHS1* | YNL192w | Chitin synthase I, requires activation from zymogenic form in order to catalyze the transfer of N-acetylglucosamine (GlcNAc) to chitin; required for repairing the chitin septum during cytokinesis; transcription activated by mating factor |  |  |
|  | YLR194c | Structural constituent of the cell wall attached to the plasma membrane by a GPI-anchor; expression is upregulated in response to cell wall stress |  | **Yes** |
| *KRE6* | YPR159w | Type II integral membrane protein required for beta-1,6 glucan biosynthesis; putative beta-glucan synthase; localizes to the ER, plasma membrane, sites of polarized growth and secretory vesicles; functionally redundant with Skn1p |  |  |
| *MNN9* | YPL050c | Subunit of Golgi mannosyltransferase complex also containing Anp1p, Mnn10p, Mnn11p, and Hoc1p that mediates elongation of the polysaccharide mannan backbone; forms a separate complex with Van1p that is also involved in backbone elongation |  | **Yes** |
| *GAS1* | YMR307w | Beta-1,3-glucanosyltransferase, required for cell wall assembly and also has a role in transcriptional silencing; localizes to the cell surface via a glycosylphosphatidylinositol (GPI) anchor; also found at the nuclear periphery |  |  |
| *HSP150* | YJL159w | O-mannosylated heat shock protein that is secreted and covalently attached to the cell wall via beta-1,3-glucan and disulfide bridges; required for cell wall stability; induced by heat shock, oxidative stress, and nitrogen limitation | **Yes** |  |
| *PKH1* | YDR490c | Serine/threonine protein kinase involved in sphingolipid-mediated signaling pathway that controls endocytosis; activates Ypk1p and Ykr2p, components of signaling cascade required for maintenance of cell wall integrity; redundant with Pkh2p |  |  |

## ^a^ Described in the *Saccharomyces* Genome Database ([**http://www.yeastgenome.org/**](http://www.yeastgenome.org/))

## ^b^ According to YEASTRACT ([**http://www.yeastract.com/**](http://www.yeastract.com/))

## ^c^ According to The *Saccharomyces cerevisiae* Promoter Database (http://rulai.cshl.edu/SCPD)
