# Supplementary material for: The resistance of the yeast Saccharomyces cerevisiae to the biocide polyhexamethylene biguanide: involvement of cell wall integrity pathway and emerging role for YAP1
Source: BMC Mol Biol. 2011 Aug 19;12:38. doi: 10.1186/1471-2199-12-38 (PMC3175164; doi:10.1186/1471-2199-12-38)
Supplement: Additional file 2 — Mutant strains with deletion in genes involved in the Cell Wall Integrity (CWI) mechanism. List of BY4741-derivative mutant strains with deletion in genes involved in the Cell Wall Integrity (CWI) mechanism, including genes belonging to PKC1 and HOG pathways. The presence of general stress DNA binding motifs (STRE) and the recognition sequence for the transcription factor Yap1p (YRE) in the promoter region of those genes are shown. [file 1471-2199-12-38-S2.DOC]

**Additional file 2**

| **Euroscarf Access number deletion strain** | **Mutated gene** | | **Function of gene^a^** | **STRE motif ^b^** | **YRE motif ^c^** |
| --- | --- | --- | --- | --- | --- |
| **Y06255** | ***wsc3*** | Partially redundant sensor-transducer of the stress-activated PKC1-MPK1 signaling pathway involved in maintenance of cell wall integrity; involved in the response to heat shock and other stressors; regulates 1,3-beta-glucan synthesis | | **Yes** |  |
| **Y01153** | ***mid1*** | O-glycosylated plasma membrane protein that acts as a sensor for cell wall integrity signaling and activates the pathway; interacts with Rom2p, a guanine nucleotide exchange factor for Rho1p, and with cell integrity pathway protein Zeo1p | |  |  |
| **Y01161** | ***wsc2*** | Partially redundant sensor-transducer of the stress-activated PKC1-MPK1 signaling pathway involved in maintenance of cell wall integrity and recovery from heat shock; secretory pathway Wsc2p is required for the arrest of secretion response | |  |  |
| **Y01784** | ***slg1*** | Sensor-transducer of the stress-activated PKC1-MPK1 kinase pathway involved in maintenance of cell wall integrity; involved in organization of the actin cytoskeleton; secretory pathway Wsc1p is required for the arrest of secretion response | | **Yes** |  |
| **Y05241** | ***mid2*** | O-glycosylated plasma membrane protein that acts as a sensor for cell wall integrity signaling and activates the pathway; interacts with Rom2p, a guanine nucleotide exchange factor for Rho1p, and with cell integrity pathway protein Zeo1p | |  |  |
| **Y05280** | ***rom2*** | GDP/GTP exchange protein (GEP) for Rho1p and Rho2p; mutations are synthetically lethal with mutations in rom1, which also encodes a GEP | |  |  |
| **Y02487** | ***mkk1*** | Mitogen-activated kinase kinase involved in protein kinase C signaling pathway that controls cell integrity; upon activation by Bck1p phosphorylates downstream target, Slt2p; functionally redundant with Mkk2p | | **Yes** |  |
| **Y01328** | ***bck1*** | Mitogen-activated protein (MAP) kinase kinase kinase acting in the protein kinase C signaling pathway, which controls cell integrity; upon activation by Pkc1p phosphorylates downstream kinases Mkk1p and Mkk2p | | **Yes** |  |
| **Y02112** | ***mkk2*** | Mitogen-activated kinase kinase involved in protein kinase C signaling pathway that controls cell integrity; upon activation by Bck1p phosphorylates downstream target, Slt2p; functionally redundant with Mkk1p | | **Yes** |  |
| **Y00993** | ***slt2*** | Serine/threonine MAP kinase involved in regulating the maintenance of cell wall integrity, progression through the cell cycle, and nuclear mRNA retention in heat shock; regulated by the PKC1-mediated signaling pathway | |  |  |
| **Y02739** | ***rlm1*** | MADS-box transcription factor, component of the protein kinase C-mediated MAP kinase pathway involved in the maintenance of cell integrity; phosphorylated and activated by the MAP-kinase Slt2p | |  |  |
| **Y06109** | ***swi4*** | DNA binding component of the SBF complex (Swi4p-Swi6p), a transcriptional activator that in concert with MBF (Mbp1-Swi6p) regulates late G1-specific transcription of targets including cyclins and genes required for DNA synthesis and repair | |  |  |
| **Y04131** | ***swi6*** | Transcription cofactor, forms complexes with Swi4p and Mbp1p to regulate transcription at the G1/S transition; involved in meiotic gene expression; cell wall stress induces phosphorylation by Mpk1p, which regulates Swi6p localization | |  | **Yes** |
| **Y02900** | ***skn7*** | Nuclear response regulator and transcription factor, part of a branched two-component signaling system; required for optimal induction of heat-shock genes in response to oxidative stress; involved in osmoregulation | | **Yes** | **Yes** |
| **Y05882** | ***knr4*** | Protein involved in the regulation of cell wall synthesis; proposed to be involved in coordinating cell cycle progression with cell wall integrity | |  |  |
| **Y05353** | ***crz1*** | Transcription factor that activates transcription of genes involved in stress response; nuclear localization is positively regulated by calcineurin-mediated dephosphorylation | | **Yes** |  |
| **Y06979** | ***gsc2*** | Catalytic subunit of 1,3-beta-glucan synthase, involved in formation of the inner layer of the spore wall; activity positively regulated by Rho1p and negatively by Smk1p; has similarity to an alternate catalytic subunit, Fks1p (Gsc1p) | |  |  |
| **Y01266** | ***hsp150*** | O-mannosylated heat shock protein that is secreted and covalently attached to the cell wall via beta-1,3-glucan and disulfide bridges; required for cell wall stability; induced by heat shock, oxidative stress, and nitrogen limitation | | **Yes** |  |
| **Y07101** | ***pbs2*** | MAP kinase kinase that plays a pivotal role in the osmosensing signal-transduction pathway, activated under severe osmotic stress; plays a role in regulating Ty1 transposition | |  |  |
| **Y02724** | ***hog1*** | Mitogen-activated protein kinase involved in osmoregulation. via three independent osmosensors; mediates the recruitment and activation of RNA Pol II at Hot1p-dependent promoters; localization regulated by Ptp2p and Ptp3p | |  |  |
| **Y05271** | ***ste11*** | Signal transducing MEK kinase involved in pheromone response and pseudohyphal/invasive growth pathways where it phosphorylates Ste7p, and the high osmolarity response pathway, via phosphorylation of Pbs2p; regulated by Ste20p and Ste50p | |  |  |
| **Y3265** | ***tps1*** | Synthase subunit of trehalose-6-phosphate synthase/phosphatase complex, which synthesizes the storage carbohydrate trehalose; also found in a monomeric form; expression is induced by the stress response and repressed by the Ras-cAMP pathway | | **Yes** |  |

## ^a^ Described in the *Saccharomyces* Genome Database ([**http://www.yeastgenome.org/**](http://www.yeastgenome.org/))

## ^b^ According to YEASTRACT ([**http://www.yeastract.com/**](http://www.yeastract.com/))

## ^c^ According to The *Saccharomyces cerevisiae* Promoter Database (http://rulai.cshl.edu/SCPD)
