# Supplementary material for: The resistance of the yeast Saccharomyces cerevisiae to the biocide polyhexamethylene biguanide: involvement of cell wall integrity pathway and emerging role for YAP1
Source: BMC Mol Biol. 2011 Aug 19;12:38. doi: 10.1186/1471-2199-12-38 (PMC3175164; doi:10.1186/1471-2199-12-38)
Supplement: Additional file 3 — Relative quantity of reference genes. Relative quantity of reference genes in the presence (test sample) or absence (reference sample) of PHMB and heat shock (HS) used as input data for geNorm analysis. [file 1471-2199-12-38-S3.DOC]

**Additional file 3**

| Strain | Condition | *PDC1* | *LEU 4* | *ADK1* | *ADH3* | *EFB1* |
| --- | --- | --- | --- | --- | --- | --- |
| BY4742 | PHMB- | 1 | 0.763842 | 0.890362 | 0.610649 | 1 |
|  | PHMB+ | 0.54050112 | 0.567732 | 1 | 0.598391 | 0.928028 |
| BY4742 | HS- | 0.5784699 | 0.810724 | 0.648417 | 0.570441 | 0.885697 |
|  | HS+ | 0.77111866 | 0.58069 | 0.395711 | 0.511101 | 0.571733 |
| *Δyap1* | PHMB- | 0.74531293 | 1 | 0.480728 | 0.456909 | 0.628733 |
|  | PHMB+ | 0.73768906 | 0.747668 | 0.496117 | 0.450678 | 0.585818 |
| *Δyap1* | HS- | 0.81905395 | 0.823948 | 0.71101 | 0.550692 | 0.71354 |
|  | HS+ | 0.78110481 | 0.731537 | 0.69737 | 0.507337 | 0.662758 |
| JP1 | PHMB- | 0.79166184 | 0.782538 | 0.595632 | 0.398014 | 0.665396 |
|  | PHMB+ | 0.92110363 | 0.874388 | 0.768396 | 0.313645 | 0.784404 |
| PE-2 | PHMB- | 0.83063163 | 0.840861 | 0.683017 | 0.327287 | 0.735036 |
|  | PHMB+ | 0.83034781 | 0.731263 | 0.668149 | 1 | 0.903826 |
|  |  |  |  |  |  |  |
