# Supplementary figures and images for: The resistance of the yeast Saccharomyces cerevisiae to the biocide polyhexamethylene biguanide: involvement of cell wall integrity pathway and emerging role for YAP1
Source: BMC Mol Biol. 2011 Aug 19;12:38. doi: 10.1186/1471-2199-12-38 (PMC3175164; doi:10.1186/1471-2199-12-38)

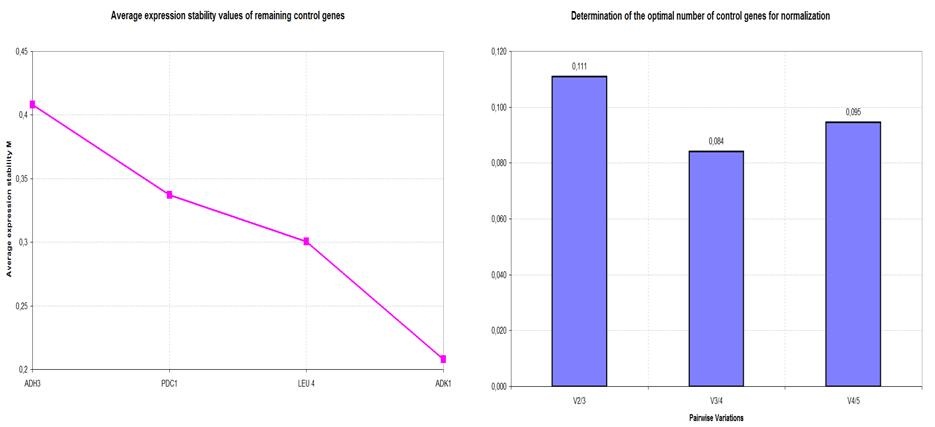

Supplement: Additional file 4 — Selection of reference genes for RT-qPCR. Graphics of Average expression stability values of remaining control genes and Determination of the optimal number of control genes for normalization obtained from geNorm analysis of the reference genes tested. [file 1471-2199-12-38-S4.JPEG]
