# Supplementary material for: The resistance of the yeast Saccharomyces cerevisiae to the biocide polyhexamethylene biguanide: involvement of cell wall integrity pathway and emerging role for YAP1
Source: BMC Mol Biol. 2011 Aug 19;12:38. doi: 10.1186/1471-2199-12-38 (PMC3175164; doi:10.1186/1471-2199-12-38)
Supplement: Additional file 5 — Normalization factors calculation for RT-qPCR analysis. Summary of the normalization factors calculation of the reference genes ADK1 and EFB1 for the biological replicates (experiments 1 and 2) in the presence (test sample) or absence (reference sample) of PHMB and heat shock (HS) used as input data for geNorm analysis. [file 1471-2199-12-38-S5.DOC]

**Additional file 5**

| Strain | Condition | *ADK1* | |  |  |  |  | *EFB1* | |  |  |  |  |  |  |
| --- | --- | --- | --- | --- | --- | --- | --- | --- | --- | --- | --- | --- | --- | --- | --- |
|  |  | Exp. 1 | Exp. 2 | mean Cq | SD Cq | Quantity | SD (±) | Exp.1 | Exp.2 | mean Cq | SD Cq | Quantity | SD (±) | **NF** | SD (±) |
| BY4741 | PHMB- | 18.35097 | 18.02074 | 18.18586 | 0.233513 | 0.890362 | 0.144113 | 17.52297 | 16.94674 | 17.23486 | 0.407454 | 1 | 0.282426 | **0.945181** | 0.139662 |
|  | PHMB+ | 18.27903 | 17.75761 | 18.01832 | 0.368706 | 1 | 0.255568 | 17.50957 | 17.17566 | 17.34262 | 0.236115 | 0.928028 | 0.151883 | **0.964014** | 0.094627 |
|  | HS- | 18.9375 | 18.34915 | 18.64333 | 0.416023 | 0.648417 | 0.186981 | 17.70078 | 17.11917 | 17.40997 | 0.41126 | 0.885697 | 0.25248 | **0.767057** | 0.125276 |
|  | HS+ | 19.62279 | 19.08881 | 19.3558 | 0.377577 | 0.395711 | 0.103564 | 17.58222 | 18.50067 | 18.04144 | 0.64944 | 0.571733 | 0.25737 | **0.483722** | 0.117159 |
| *Δyap1* | PHMB- | 18.73549 | 19.41456 | 19.07503 | 0.480175 | 0.480728 | 0.160002 | 17.39806 | 18.41061 | 17.90434 | 0.715984 | 0.628733 | 0.312029 | **0.554731** | 0.153014 |
|  | PHMB+ | 19.48673 | 18.57241 | 19.02957 | 0.646522 | 0.496117 | 0.222327 | 17.61759 | 18.39507 | 18.00633 | 0.549763 | 0.585818 | 0.223236 | **0.540968** | 0.130232 |
|  | HS- | 18.73578 | 18.28497 | 18.51038 | 0.31877 | 0.71101 | 0.157101 | 17.87743 | 17.56615 | 17.72179 | 0.220113 | 0.71354 | 0.108865 | **0.712275** | 0.06303 |
|  | HS+ | 18.70422 | 18.37242 | 18.53832 | 0.23462 | 0.69737 | 0.11341 | 17.64906 | 18.00755 | 17.8283 | 0.253492 | 0.662758 | 0.116452 | **0.680064** | 0.064243 |
| JP1 | PHMB- | 18.58486 | 18.9468 | 18.76583 | 0.255931 | 0.595632 | 0.105664 | 17.95183 | 17.69331 | 17.82257 | 0.182796 | 0.665396 | 0.084309 | **0.630514** | 0.044905 |
|  | PHMB+ | 18.52684 | 18.26996 | 18.3984 | 0.181644 | 0.768396 | 0.096746 | 17.79171 | 17.37866 | 17.58519 | 0.292074 | 0.784404 | 0.158803 | **0.776400** | 0.081668 |
| PE-2 | PHMB- | 18.71553 | 18.42112 | 18.56833 | 0.20818 | 0.683017 | 0.098559 | 17.54812 | 17.80982 | 17.67897 | 0.185049 | 0.735036 | 0.09428 | **0.709026** | 0.049163 |
|  | PHMB+ | 18.81469 | 18.38547 | 18.60008 | 0.303504 | 0.668149 | 0.140561 | 17.1496 | 17.61188 | 17.38074 | 0.326885 | 0.903826 | 0.204788 | **0.785987** | 0.09774 |
